# PARylation-mediated post-transcriptional modifications in cancer immunity and immunotherapy

**DOI:** 10.3389/fimmu.2025.1537615

**Published:** 2025-03-11

**Authors:** Kazuya Matsumoto, Yoshinori Matsumoto, Jun Wada

**Affiliations:** Department of Nephrology, Rheumatology, Endocrinology and Metabolism, Faculty of Medicine, Dentistry and Pharmaceutical Sciences, Okayama University, Okayama, Japan

**Keywords:** PARylation, cancer, post-transcriptional regulation, ubiquitylation, immune system

## Abstract

Poly-ADP-ribosylation (PARylation) is a post-translational modification in which ADP-ribose is added to substrate proteins. PARylation is mediated by a superfamily of ADP-ribosyl transferases known as PARPs and influences a wide range of cellular functions, including genome integrity maintenance, and the regulation of proliferation and differentiation. We and others have recently reported that PARylation of SH3 domain-binding protein 2 (3BP2) plays a role in bone metabolism, immune system regulation, and cytokine production. Additionally, PARylation has recently gained attention as a target for cancer treatment. In this review, we provide an overview of PARylation, its involvement in several signaling pathways related to cancer immunity, and the potential of combination therapies with PARP inhibitors and immune checkpoint inhibitors.

## Introduction

Poly-ADP-ribosylation (PARylation) is a post-translational modification in which ADP-ribose is added to substrate proteins. PARylation is mediated by a superfamily of ADP-ribosyl transferases known as PARPs and has a wide range of effects on cellular functions, including proliferation and differentiation. Additionally, PARylation-mediated post-transcriptional modifications have recently gained attention as targets for cancer treatment. In this review, we provide an overview of PARylation and its involvement in several signaling pathways related to cancer immunity. Lastly, we will discuss the relationship between PARylation and immune checkpoint inhibitors.

## ADP-ribosylation

ADP-ribosylation is a reversible post-translational modification that is required for regulation of molecular interactions (1, 2). During ADP-ribosylation, nicotinamide adenine dinucleotide (NAD^+^) is consumed as a donor and split into ADP-ribose and nicotinamide (NAM), resulting in addition of ADP-ribose to a substrate (2, 3). The addition of a single ADP-ribose is referred to as mono-ADP ribosylation (MARylation). In contrast, the reaction that adds two or more ADP-ribose units or creates a branched structure is called poly-ADP-ribosylation (PARylation) (4, 5). PARylation primarily affects proteins, although previous reports have shown that it can also modify nucleic acids (6, 7).

ADP-ribosylation is mediated by the ADP-ribosyl transferase superfamily (ARTs), which comprises 23 families including diphtheria toxin-like ARTs (ARTDs) and cholera toxin-like ARTs (ARTCs). ARTDs consist of 17 members, referred to as PARPs (PARP1-PARP16), with PARP5A and PARP5B also called tankyrase 1 (TNKS1) and tankyrase 2 (TNKS2), respectively (2, 4, 5, 8, 9). Among the ARTD members, only PARP1, PARP2, TNKS1, and TNKS2 exhibit PARylation activity, and they are referred to as poly-ARTs, despite the name PARP historically being derived from poly ADP-ribose polymerase. In contrast, the other members (PARP3, PARP4, PARP6-12, PARP14-16) possess MARylation activity and are referred to as mono-ARTs. PARP13 is thought to be inactive due to a defect in its NAD^+^ binding residues (5, 10).

ADP-ribosylation by “writers” such as PARPs is recognized by “reader” proteins that contain specific modules or motifs, including *macro* domains, PAR binding zinc finger (PBZ) domains, WWE domains, and the PAR-binding motif (9, 11, 12). Ubiquitin E3 ligase ring finger protein 146 (RNF146) is a reader protein in which the WWE domain detects PARylation by binding to the iso-ADP-ribose moiety (13, 14). ADP-ribosylation is quickly terminated by the removal of ADP-ribose by “eraser” proteins such as poly(ADP-ribose) glycohydrolase (PARG), MacroD1, MacroD2, terminal ADP-ribose protein glycohydrolase 1 (TARG1), and the ADP-ribose hydrolase (ARH) members ARH1 and ARH3 (15–17).

PARylation regulates a wide range of molecular functions, including transcription, RNA regulation, mitosis, telomere length maintenance, cell-cycle regulation, cellular differentiation, DNA damage response, protein degradation, ubiquitination, metabolism, and innate and adaptive immunity, among many others (3, 15, 18–25) (**Table 1**).

**Table 1:** Physiological functions of PARylation-mediated modification of proteins.

Classification of function	PARP protein	Associated functions
Transcription	PARP1	Chromatin remodeling (26–28) Regulation of transcription through directly binding to various promoters (29) Promotion of transcription through activation of the transcription of DNA methyltransferase 1 (DNMT1) (30)
RNA regulation	PARP1	Splicing regulation through interaction with heterogeneous nuclear-ribonucleoproteins (hnRNPs), including A1, A2/B1, C1/C2, G, H, K, M, E1 (31), hrp38 (32), splicing factors, including splicing factor/splicing factor 2 (ASF/SF2) (33), SF3B1, SF3A1 and SF3B2 (34) Inhibition of polyadenylation via PARylation of poly (A) polymerase in response to heat shock (35)
Translation	PARP1	Regulation of translation of E-cadherin through PARylation of hnRNPs (36–38)
Mitosis	Tankyrase1 Tankyrase2	Regulation of mitotic spindle via PARylation on NuMA (39, 40) Regulation of formation and function of centrosome through PARylation of Miki (41) and CPAP (42)
Telomere length maintenance	PARP1	Regulation of telomerase enzymes and alternative lengthening of telomeres (ALT) (43) Restoration of double strand breaks (DSBs) of telomeres through alternative end-joining (Alt-EJ) (43)
	Tankyrase1 Tankyrase2	Telomere elongation via PARylation on TRF1 (44) Regulation of telomere segregation during mitosis through degradation of cohesin (45)
DNA damage response	PARP1 PARP2	Single strand break (SSB) repair through base excision repair (BER) and nucleotide excision repair (NER) (46, 47) DSB repair through homologous recombination (HR), nonhomologous end-joining (NEHJ), and alt-EJ (25, 47, 48)
Cellular differentiation and development	PARP1 PARP2	Regulation of T cell differentiation (49–53), B cell development (54), and dendritic cell maturation (55)
Proteasomal degradation and signal transduction	Tankyrase1	Regulation of Wnt/β-catenin pathway (56), Hippo pathway (57), PI3K/Akt pathway (58) and LKB1/MAPK pathway (59) Modulation of proteasome activity via PARylation of PI31 (60)
Innate immunity	PARP1	Activation of the NK-κB pathway (61–64) Release of the high-mobility group box 1 (HMGB1) from the nucleus to cytoplasm in macrophages (65–67) Activation of cGAS-cGAMP-STING and subsequent type I IFN release (68) Regulation of neutrophil recruitment (69)
Metabolism	PARP1 PARP2	Regulation of NAD+ metabolism (70), mitochondrial activity (71, 72), glucose metabolism (73–77), and lipid metabolism (23, 71, 78–83)
	Tankyrase1 Tankyrase2	Regulation of glucose metabolism via GLUT4 translocation and insulin release (84–86)

## PARylation modulates DNA damage response and transcription

DNA is constantly exposed to endogenous and exogenous damage, requiring frequent restoration to maintain genome integrity (87). PARP1, an abundant nuclear protein, plays a crucial role in the early phase of DNA damage response (DDR). When single-strand breaks (SSBs) are detected by PARP1 or PARP2, PARylation occurs on PARP1 (self-PARylation) or DDR-associated proteins (25, 46). PAR recruits the scaffold protein XRCC1 and its partner proteins, facilitating the repair of SSBs (46). Both PARylation on PARP1 and histones induces chromatin decompaction, thereby promoting transcription (26, 27). In response to double-strand breaks (DSBs), homologous recombination (HR) and non-homologous end-joining (NHEJ) are the primary mechanisms for repair. During HR, activated PARP1 recruits the MRE11-RAD50-NBS1 complex to the sites of damage (25). PARP1 inhibits the classical pathway of NHEJ by binding to DSBs in direct competition with Ku70/80 proteins and promotes alternative NHEJ (Alt-EJ) by recruiting MRN and CtIP (25, 48). Since the accumulation of DNA damage contributes to the pathophysiology of tumorigenesis, neurodegeneration, and premature aging, DDR is a critical mechanism for preventing these diseases (47).

Nuclear stress, such as heat shock, activates PARP1, leading to the PARylation of poly(A) polymerase (PAP). This modification prevents PAP from binding to target mRNA and inhibits subsequent 3’ mRNA processing, resulting in decreased mRNA synthesis (35). Additionally, splicing is regulated by PARP1-mediated PARylation of heterogeneous nuclear ribonucleoproteins (hnRNPs) hrp38 and squid in *Drosophila* (32).

## Role of PARylation in mitosis and telomere length maintenance

In cell division, mitotic spindle formation is a crucial mechanism for the segregation of chromosomes into two daughter cells (88). Spindle orientation is determined by Gαi-LGN-NuMA complex, which regulates the extent of microtubule-pulling forces (89). It has been reported that NuMA localizes tankyrase1 to spindle poles and that tankyrase1 PARylates NuMA at the onset of mitosis (39, 40). Miki, another protein associated with mitosis, has also been shown to undergo PARylation by tankyrase1 during late G2 and prophase. This modification translocates Miki to mitotic centrosomes from the Golgi apparatus, anchoring CG-NAP, which serves as a scaffold for the γ-tubulin ring complex. Tankyrase1 knockdown impairs spindle formation and causes mitotic defects in prometaphase, such as preanaphase arrest, chromosome scattering, and pseudometaphase, highlighting the importance of PARylation in normal cell division (39–41).

Telomeres, nucleoprotein structures located at the ends of chromosomes, play a key role in maintaining genome integrity (90, 91). Chromosome duplication presents intrinsic challenges, including the inability of DNA polymerases to fully replicate the ends of chromosomes and the misrecognition of chromosome ends as DSBs, leading to improper repair. Telomeres address these issues and prevent genome instability by protecting chromosome ends (90, 91).

Telomeres are associated with a six-subunit protein complex called Shelterin, which consists of TRF1, TRF2, Rap1, TIN2, TPP1, and POT1 (92). TRF1 negatively regulates telomerase activity by limiting its accessibility for DNA (93, 94). Tankyrase-mediated PARylation of TRF1 inhibits the binding between TRF1 and DNA, allowing telomerase to extend telomeres (44, 95). TIN2 forms a ternary complex with TRF1 and tankyrase, repressing PARylation on TRF1 (96). Since telomeres harbor PARP1 activation sites, PARP1 is considered a potential inhibitor of telomere activity (92). TRF2 and TIN2 have been reported to protect telomeres from PARP1 independently (97).

## PARylation regulates the ubiquitin-proteasome system

The ubiquitin-proteasome system (UPS) is a pivotal mechanism that controls the stability of intracellular proteins, modulating processes such as the cell cycle, apoptosis, transcription, and protein quality control (98, 99). Three classes of enzymes, ubiquitin-activating enzymes (E1), ubiquitin-conjugating enzymes (E2), and ubiquitin ligases (E3), facilitate the addition of ubiquitin chains to substrate proteins, which are subsequently recognized and degraded by the proteasome into peptide chains (99). Since numerous proteins, including oncoproteins and tumor suppressor proteins, are regulated by the UPS, abnormal ubiquitination has been reported to contribute to the development of various cancers (98, 100).

PI31 is an evolutionarily conserved protein that was initially identified as a suppressor of proteasome (101); however, subsequent studies have also suggested that PI31 activates the 20S core protease (102, 103). PI31 undergoes tankyrase-mediated PARylation, which decreases its affinity for 20S proteasome α-subunits, thereby reducing the inhibitory effect of PI31 (60). This modification enhances the binding and sequestration of dp27 and dS5b from 19S regulatory particles, promoting 26S proteasome assembly (60). In summary, tankyrase modulates proteasome activity through PARylation of PI31.

The well-known role of tankyrase is PARylation-mediated proteasomal degradation. Tankyrase PARylates substrates such as AXIN, PTEN, TRF1, RNF146, 3BP2, BLZF1, and CASC3 (104, 105). The E3-ubiquitin ligase RNF146 recognizes these PARylation modifications and ubiquitinates the substrate proteins, leading to their proteasomal degradation (104, 105). The significance of PARylation-mediated proteasomal degradation in several signaling pathways will be discussed in later sections.

## PARylation as a modulator of innate immunity

It has been reported that PARP inhibitors (PARPis) reduced the transcription and release of lipopolysaccharide (LPS)-induced inflammation mediators, including TNF-α, IL-1, IL-6, and nitrite (NO_2_^-^), in murine bone marrow-derived macrophages (106). Additionally, macrophages derived from PARP1-deficient mice showed defective nuclear factor kappa B (NK-κB) activation and decreased production of TNF-α and IFN-γ in response to LPS (61). These PARP1-deficient mice were protected from death due to septic shock, highlighting the importance of PARP1 as an inflammatory mediator (61). In a zymosan-induced peritonitis model, a previous study showed that inhibition of poly (ADP-ribosyl) synthetase (PARS) suppressed neutrophil recruitment to sites of inflammation through postcapillary venules, providing protection against organ injury (69). PARP1 forms a complex with the subunits of NF-κB (p50 and p65 (RelA)), a key regulator of transcription involved in immune response and inflammation (107). The interaction between PARP1 and p65 was shown to be essential for NF-κB-dependent transcription of the iNOS and P-selectin promoters in B and T cells (107). In the inflammatory state induced by LPS, extracellular signal-regulated kinases 1/2 (ERK1/2) directly activate PARP1 through phosphorylation at serine 372 or threonine 373 (62), inducing PARylation-mediated activation of p65 and subsequent transcriptions of proinflammatory genes (63). The non-receptor tyrosine kinase c-Abl also activates PARP1 via phosphorylation at tyrosine 829, resulting in PARylation of p65 (64). Notably, extracellular PAR is recognized by Toll-like receptor 2 (TLR2) or TLR4 on macrophages, triggering cytokine release in humans and mice, suggesting that PAR may function as a damage-associated molecular pattern (DAMP) (108).

## Tankyrase regulates bone metabolism and the immune system via PARylation-mediated proteasomal degradation of 3BP2

SH3 domain-binding protein-2 (3BP2) was initially identified as a protein binding to the Src homology 3 (SH3) domain of Abl (109, 110). Subsequent studies revealed that 3BP2 functions as an adaptor protein, forming a signaling complex with SYK (111), SRC (112, 113), and VAV (114), thereby regulating intracellular signaling pathways. Gain-of-function missense mutations in the *SH3BP2* gene have been identified as the cause of cherubism, which is an autosomal dominant disorder characterized by facial swelling owing to severe craniofacial bone destruction and subsequent fibrous tissue masses (115, 116). Cherubism model mice with a mutation in the *Sh3bp2* gene exhibit hyperactivity of macrophages and osteoclasts, leading to systemic inflammation and bone loss (116, 117).

**Figure 1:**
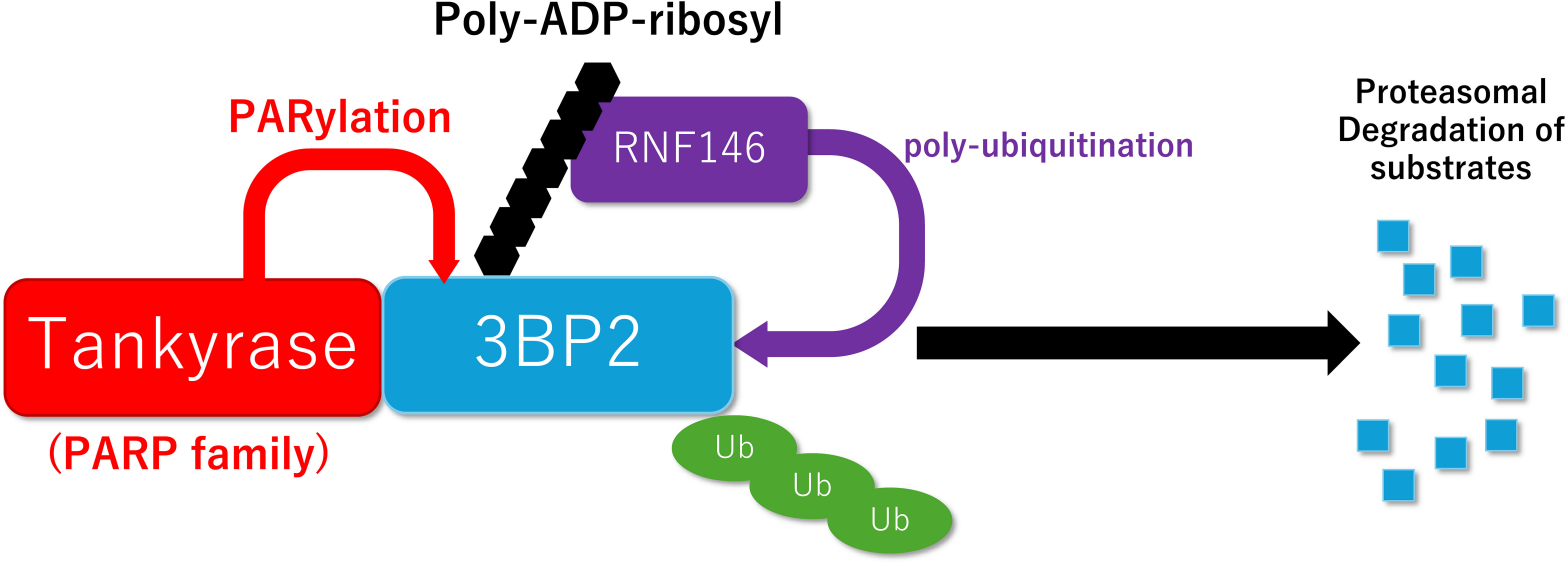
Schematic model of PARylation-mediated protein degradation. Tankyrase promotes poly-ADP-ribosylation (PARylation) of 3BP2, which creates a recognition site for RNF146, leading to ubiquitylation and subsequent proteasomal degradation of 3BP2.

Prof. Robert Rottapel’s lab at the University of Toronto, Canada has provided mechanistic insights by demonstrating that the gain-of-function mutations protect 3BP2 from tankyrase-mediated PARylation and proteasomal degradation (118, 119) (**Figure 1**). The missense mutation uncouples 3BP2 from the proteasomal degradation, leading to its accumulation in cells and hyperactivation of its substrates SYK, SRC, and VAV. Consequently, the loss of tankyrase-mediated degradation of 3BP2 underlies the pathophysiology of cherubism (118).

Furthermore, they and we have discovered that 3BP2-induced activation of ABL and SRC is required for both RUNX2-mediated osteoblastogenesis and NFATc1-mediated osteoclastogenesis, respectively (112, 120). We have shown that conditional knockout of *Rnf146* leads to stabilization of AXIN in osteoblasts and osteoclasts, resulting in phenotypes resembling osteoporosis and cleidocranial dysplasia (CCD), respectively (121, 122). Tankyrase regulates the TLR signaling pathways via PARylation-mediated degradation of 3BP2, and dysregulation of 3BP2 leads to autoinflammatory phenotypes, including severe inflammatory bowel disease (123, 124). 3BP2 is also required for proliferation and activation of T cells (125) and B cells (126) as well as for optimal neutrophil chemoattractant responses and host defense (127). Altogether, PARylation-mediated degradation of 3BP2 is a crucial mechanism for regulation of bone metabolism and the immune system (128–131).

## Wnt/β-catenin signaling pathway

Wnt/β-catenin signaling is involved in embryonic development and cell homeostasis (132–134). The Wnt signaling pathway is primarily divided into three pathways: the canonical, β-catenin-dependent pathway and the non-canonical Wnt/Ca^2+^ (calcium) and Wnt/PCP (planar cell polarity) pathways (135). AXIN negatively regulates the canonical pathway by acting as a scaffold protein that forms the destruction complex (DC), which includes the tumor suppressor protein APC and the two serine-threonine kinases CK1α/δ and GSK3α/β (134).

Dysregulation of the Wnt/β-catenin pathway is associated with various types of cancer, including colorectal cancer (136), hepatocellular carcinoma (137), cholangiocarcinoma (138), lung cancer (139), hematological malignancies (140), and melanoma (141). As a result, the Wnt/β-catenin pathway has emerged as a potential target for cancer therapy (134, 142, 143), although such therapies are not yet in practical use.

Huang et al. reported that the tankyrase inhibitor XAV939 stabilizes AXIN and inhibits the Wnt/β-catenin pathway. They revealed that AXIN binds to tankyrase in the tankyrase-binding domain (TBD) and undergoes PARylation and ubiquitination (56). In addition to inducing proteolysis, tankyrase promotes accumulation of AXIN in the stimulatory signalosome and enhances the interaction between AXIN and the Wnt co-receptor LRP6 in response to Wnt stimulation (144). Tankyrase can also promote Wnt/β-catenin signaling in a manner independent of its PARP catalytic activity. Crystal structure analysis of tankyrase revealed that tankyrase polymerizes on its sterile alpha motif (SAM) domains, which are required for both tankyrase-dependent Wnt signaling and intact PARylation activity (145–147). Several *in vitro* studies have shown that XAV939 inhibits the Wnt/β-catenin signaling pathway and suppresses the proliferation of various types of cancer (56, 148–150). Additionally, multiple reports suggest that upregulation of the Wnt/β-catenin pathway contributes to resistance to a PARPi (151, 152), and combined therapies targeting both PARP and the Wnt/β-catenin pathway have demonstrated a synergistic effect (151–153).

## PI3K-AKT signaling pathway

The phosphatidylinositol 3-kinase (PI3K)-AKT signaling pathway promotes cell survival, growth, differentiation, proliferation and glucose homeostasis in response to various stimuli (154). When membrane receptors, including receptor tyrosine kinases (RTKs) and G-protein-coupled receptors (GPCRs), receive extracellular signals, class I PI3K catalyzes the phosphorylation of phosphatidylinositol-bisphosphate (PIP2) to generate phosphatidylinositol-3,4,5-triphosphate (PIP3), which activates AKT and various types of AKT-dependent downstream signaling. Conversely, PTEN negatively regulates this pathway by dephosphorylating PIP3, converting it back to PIP2 (155). Upregulation of PI3K and downregulation of PTEN are recognized as tumorigenic (156, 157). In fact, a meta-analysis of cancer genome sequencing studies revealed that *PIK3CA* (which encodes one of the class1 PIK3 isoforms) and *PTEN* were the second and third most frequently mutated genes in human cancers (158).

**Figure 2:**
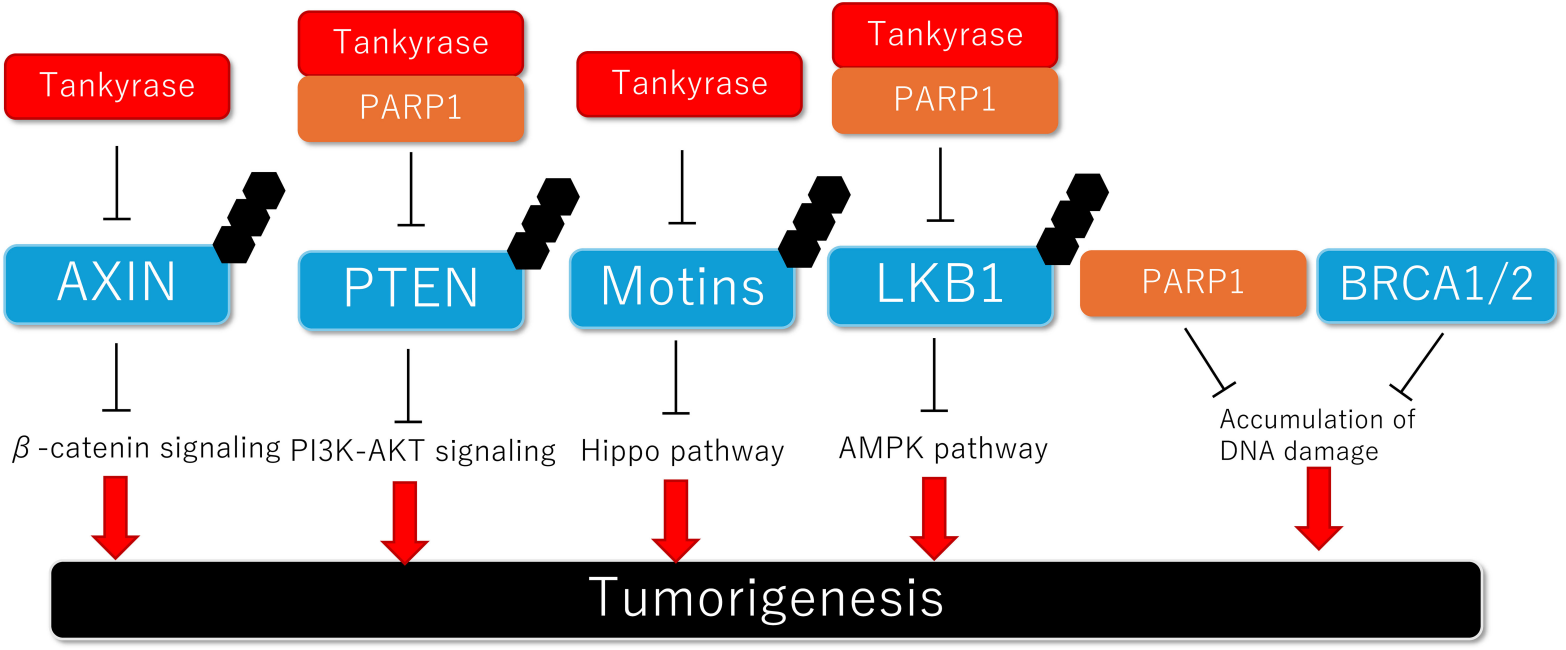
Schematic model of the association between PARylation-mediated protein degradation and tumorigenesis.

PTEN has been reported to be regulated by PARylation-mediated degradation (**Figure 2**). Double knockdown of tankyrase 1/2 stabilized PTEN and downregulated AKT signaling, leading to the suppression of colon carcinoma proliferation (58). Additionally, investigation of human colon carcinoma samples revealed that tankyrase was upregulated and negatively correlated with PTEN expression (58). PARP1 also indirectly inhibits PTEN expression through PARylation-mediated degradation of its master regulator, Snail. Inhibition of PARP1 prevents doxorubicin-induced PTEN suppression, suggesting that combined therapy with a PARP1 inhibitor and cytotoxic drugs could be a promising treatment strategy (159, 160). Moreover, a recent study showed that AKT activation induces nuclear localization of glutamyl-prolyl-tRNA synthetase (EPRS1), which binds to PARP1 and activates PARylation, thereby contributing to breast cancer cell survival (161). Some preclinical studies have shown that PI3K inhibition enhances sensitivity to PARPis by suppressing HR repair (162, 163). In a phase I trial evaluating the efficacy of the PARPi olaparib combined with the AKT inhibitor capivasertib for advanced solid tumors, the combination therapy was well tolerated (164). Regarding efficacy, 44.6% of patients (25 out of 56 patients) had clinical benefits, including complete remission (CR), partial remission (PR), or stable disease (SD) lasting more than 4 months (164).

## Hippo pathway

The Hippo pathway is a highly conserved growth control system that regulates cell, tissue or organ growth. This system can be activated by a broad range of extracellular stimuli, including changes in tight junctions and adherence junctions, energy stress, heat shock, osmotic stress, glycogen accumulation and mechanical forces. Downstream, Yes-associated protein (YAP) and its paralog, transcriptional co-activator PDZ-binding motif (TAZ), are phosphorylated and retained in the cytoplasm, leading to repression of the pro-growth transcriptional activity (165). Dysregulation of the Hippo pathway is thought to promote tumorigenesis, although it is not a direct trigger for cancer development (166–168).

Motin family proteins (Motins), including Angiomotin (AMOT), Angiomotin like 1 (AMOTL1) and Angiomotin like 2 (AMOTL2), are known to negatively regulate YAP by retaining it in the cytosol (169–172). Wang et al. reported that tankyrase interacts with all of the Motin family proteins (AMOT, AMOTL1, and AMOTL2) and PARylates them, leading to RNF146-mediated ubiquitination and subsequent proteasomal degradation (**Figure 2**) (57). Besides, RNF166 has been found to recognize tankyrase-mediated PARylation on Motins (AMOT and AMOTL2). RNF166 interacted with AMOT more strongly than did RNF146, leading to K48-linked polyubiquitination and degradation of AMOT. Overexpression of RNF166 resulted in elevated YAP activity and colorectal cancer progression (173). Several *in vivo* studies have shown that tankyrase inhibitors stabilize Motins, thereby suppressing the oncogenic function or drug resistance mediated by YAP (57, 174, 175). In contrast, tankyrase has been shown to maintain the Crumbs complex, which regulates tight junctions and resistance to epithelial-to-mesenchymal transitions (EMT), by modulating the expression of Motins. That study suggested that tankyrase inhibition could induce cancer progression (176).

## LKB1/AMPK pathway

*LKB1* was originally identified as a tumor suppressor gene located on human chromosome 19p13 and it is responsible for Peutz-Jeghers syndrome, an autosomal dominant inherited disorder characterized by hamartomatous polyps and mucocutaneous pigmentation (177, 178) and also an increased risk for various malignancies including gastrointestinal, gynecological, colorectal, pancreatic, and lung cancers (179–181). *LKB1* encodes serine/threonine kinase LKB1, which forms a complex with STRADα and MO25 and phosphorylates AMP-activated protein kinase (AMPK) (182). LKB1 suppresses tumorigenesis through activation of AMPK (**Figure 2**), maintenance of cell polarity, and regulation of the cell cycle (183). Approximately 30% of human non-small cell lung cancers and 20% of cervical cancers harbor mutations in *LKB1* (184–186).

It was reported that tankyrases repress LKB1 activity through PARylation at Glu130/138 and promote K63-linked ubiquitination by RNF146, thereby blocking the formation of the LKB1/STRAD/MO25 complex (59). Additionally, both *in vitro* and *in vivo* studies showed that inhibition of tankyrase repressed tumorigenesis by activating LKB1 and AMPK (59), providing further evidence for the potential of tankyrase inhibitors as anti-cancer drugs.

In a study on *LKB1*-mutant lung cancer, Long et al. found that *LKB1* mutation caused deficiencies in the DNA damage repair process and hyperactivation of PARP1, leading to the PARylation of STAT1 (187). This modification inactivated STAT1 and resulted in downregulation of the interferon-gamma (IFNγ) response. Furthermore, the PARP1 inhibitor olaparib restored STAT1 phosphorylation and the IFNγ response.

## *BRCA1/2* mutation and PARP inhibitors

Breast cancer has the highest prevalence and mortality rate among malignancies in women worldwide (188). It is classified immunohistochemically based on positivity of estrogen receptor (ER), progesterone receptor (PR), and HER2 (189). The absence of these markers defines triple-negative breast cancer (TNBC), an aggressive subtype (189, 190). Approximately 5% of breast cancer patients have germline pathogenic variants in cancer disposition genes, with *BRCA1* and *BRCA2* being the major ones (191, 192). Germline mutations in *BRCA1/2* are also prevalent in ovarian, prostate and pancreatic cancers (193, 194).

In the DDR, BRCA1 and BRCA2 are crucial proteins for homologous recombination repair (HR), a process by which DNA is synthesized using a homologous DNA molecule as a template. Germline mutations in *BRCA1/2* lead to accumulation of DNA damage and subsequent tumorigenesis. Tumor cells with *BRCA1/2* mutation rely on alternative repair pathways, such as PARP1-mediated repairing (**Figure 2**). Therefore, PARPis exhibit anti-cancer effects through a mechanism known as synthetic lethality (25, 48).

In 2014, the U.S. Food and Drug Administration (FDA) and European Medicines Agency (EMA) approved the first PARPi, olaparib, as maintenance therapy for platinum-sensitive advanced ovarian cancer with germline mutations in *BRCA1/2*. Currently, four different PARPis (olaparib, talazoparib, rucaparib, and niraparib) are available for treatment of ovarian, breast, pancreatic, and prostate cancers (195–199).

Bromodomain-containing protein 7 (BRD7) is a tumor suppressor protein that regulates cell cycle progression and transcriptional regulation (200). It has been reported that PARP1 regulates BRD7 expression through PARylation-mediated ubiquitination, enhancing the survival of cancer cells. PARP1 inhibition not only suppresses cell proliferation but also sensitizes cancer cells to DNA-damaging chemotherapy, suggesting the potential for combined therapies using PARPis and chemotherapeutic drugs (201).

## Immune checkpoint inhibitors

T cell responses to antigen recognition by the T cell receptor (TCR) are regulated by a balance between co-stimulatory and inhibitory signals, also known as immune checkpoints (202). Well-studied immune checkpoints include programmed death-1 or its ligand (PD-1/PD-L1) as well as cytotoxic T-lymphocyte-associated antigen 4 (CTLA4). Immune checkpoint inhibitors (ICIs) enhance immune responses against malignancies by blocking these pathways (202).

PD-1 is expressed on activated CD4^+^ or CD8^+^ T cells, monocytes, natural killer T cells, B cells, and dendritic cells (203). PD-1 ligation dampens TCR signaling, cytokine release, and cell viability, while co-stimulation with CD28 can reverse these effects (203). PD-L1 is expressed by various cell types, including immune cells and tumor cells, in response to IFN-γ produced by activated T cells (204). Many human cancers, including breast, urothelial, ovarian, and pancreatic cancers, express tumor-associated PD-L1 (204). Binding of PD-L1 to PD-1 induces effector T cell exhaustion and immune evasion by tumor cells, leading to malignancy progression (203–205). Another immune checkpoint, CTLA4, is expressed on T cells and is essential for the function of regulatory T cells (206). CTLA4 and CD28 are homologous glycoproteins of the immunoglobulin superfamily (207) and share identical ligands, CD80 and CD86 (202). CTLA4 has a higher affinity than that of CD28 for these ligands, resulting in the inhibition of T cell activation (202). Since cancer cells exploit these checkpoints to evade host immune surveillance, ICIs act by blocking these pathways and activating the anti-tumor immunity of T cells (208). The U.S. FDA has approved three categories of ICIs: anti-CTLA4 inhibitor (ipilimumab), anti-PD-1 inhibitors (nivolumab, pembrolizumab, and cemiplimab), and anti-PD-L1 inhibitors (atezolizumab, durvalumab, and avelumab). Therapies with these ICIs are used for a wide range of malignancies including melanoma, breast cancer, non-small lung cancer, renal cell carcinoma, urothelial carcinoma, gastric cancer, colorectal cancer, and many others (209–211).

## PARP inhibitors/tankyrase inhibitors and immune checkpoint inhibitors show a synergistic effect

Despite the durable response rate of an ICI, many patients experience primary or acquired resistance (212), highlighting the need for new strategies, such as multidrug therapies. Several preclinical studies have suggested a synergistic effect between DDR inhibition and ICIs. PARP inhibition leads to the upregulation of PD-L1 and suppression of anticancer immunity, while PD-L1 blockade re-sensitizes PARPi-treated cancer cells to T cell cytotoxicity (213, 214). Mechanistically, inhibitors of DDR components, such as PARP or checkpoint kinase 1 (CHK1), increase cytosolic damaged DNA and activate the STING/TBK1/IRF3 innate immune pathway. This activation results in the upregulation of PD-L1, IFN-β, IFN-γ, and CCL5, which drives CD8^+^ T cell infiltration into tumors (215–217). Additionally, a PARPi has been reported to enhance PD-L1 expression by preventing PARP1-mediated dephosphorylation on STAT3 (218).

Tankyrase inhibition may also enhance the efficacy of ICIs. Spranger et al. classified metastatic human cutaneous melanoma samples into two groups based on T cell signatures: non-T-cell inflamed group and T-cell-inflamed group (219). Gene expression analysis revealed that the non-T-cell-inflamed group exhibited upregulation of Wnt/β-catenin signaling compared to that in the T-cell-inflamed group (219). This evidence suggests that activated Wnt/β-catenin signaling may inhibit antitumor T cell response. As mentioned above, tankyrase can activate Wnt/β-catenin signaling through PARylation-mediated degradation of AXIN, indicating that tankyrase inhibition could serve as a potential therapy to enhance the anticancer effect of T cells in combination with ICIs. In mouse models, tankyrase inhibitors (G007-LK and OM-153) have been shown to suppress Wnt/β-catenin signaling, leading to activation of T cell-mediated antitumor responses induced by PD-1 inhibition and suppression of melanoma growth (220, 221).

## Clinical trials on combined therapies

There has been an increasing number of clinical trials to evaluate the efficacy of combination therapies involving PARPis and ICIs. In breast cancer, the results of combination therapy have been inconsistent (222–224). A previous consensus paper recommended adjuvant treatment combining pembrolizumab with a PARP inhibitor for a limited population in patients with TNBC harboring BRCA1/2 mutations (197). A randomized phase Ib/II trial was carried out to compere two combination therapies for pancreatic cancers: niraparib with nivolumab and niraparib with ipilimumab (225). The rate of progression-free survival (PFS) at 6 months was higher in the latter group (20.6% vs 59.6%), although adverse events were more frequently observed in the latter group (225). Combination therapies for pancreatic cancer are currently being examined in multiple studies (226). Although PARPis are also crucial drugs for ovarian cancer, prolonged usage often leads to the development of PARPi resistance (227). The use of ICIs is an attractive strategy for overcoming the problem of PARPi resistance. A phase I/II study in which the combination of niraparib and pembrolizumab was evaluated showed promising tolerability and antitumor activity (228). The efficacy and safety of combination therapies involving PARPi and ICIs are currently being investigated in several ongoing studies (227). In contrast, combination therapies appear to be less effective in prostate cancer. In a phase Ib/II trial, patients with metastatic castration-resistant prostate cancer were treated with pembrolizumab plus olaparib (229). The median radiographic PFS (rPFS) and overall survival (OS) rates were 4.5 months and 14 months, respectively (229). In a subsequent phase III trial, this combination was compared to abiraterone or enzalutamide in patients pretreated with androgen receptor signaling inhibitors and docetaxel (230). That study was discontinued for futility due to a lack of improvement in rPFS and OS in the interim analysis, despite a higher objective response rate (17% vs. 0%) (230). The development of this combination therapy has been halted, and there are no ongoing phase III trials for prostate cancer (231).

## Conclusion

We have discussed the associations between PARylation and several signaling pathways involved in cancer generation and progression. Since PARylation regulates a wide range of cellular functions, it represents an attractive target for cancer therapy. Further research is needed to develop appropriate treatment strategies, including combination therapies.
